# Extensive CRISPR RNA modification reveals chemical compatibility and structure-activity relationships for Cas9 biochemical activity

**DOI:** 10.1093/nar/gky1214

**Published:** 2018-12-04

**Authors:** Daniel O’Reilly, Zachary J Kartje, Eman A Ageely, Elise Malek-Adamian, Maryam Habibian, Annabelle Schofield, Christopher L Barkau, Kushal J Rohilla, Lauren B DeRossett, Austin T Weigle, Masad J Damha, Keith T Gagnon

**Affiliations:** 1 Department of Chemistry, McGill University, Montreal, Quebec H3A 0B8, Canada; 2 Department of Chemistry & Biochemistry, Southern Illinois University, Carbondale, Illinois 62901, USA; 3 Department of Biochemistry & Molecular Biology, School of Medicine, Southern Illinois University, Carbondale, Illinois 62901, USA

## Abstract

CRISPR (clustered regularly interspaced short palindromic repeat) endonucleases are at the forefront of biotechnology, synthetic biology and gene editing. Methods for controlling enzyme properties promise to improve existing applications and enable new technologies. CRISPR enzymes rely on RNA cofactors to guide catalysis. Therefore, chemical modification of the guide RNA can be used to characterize structure-activity relationships within CRISPR ribonucleoprotein (RNP) enzymes and identify compatible chemistries for controlling activity. Here, we introduce chemical modifications to the sugar–phosphate backbone of *Streptococcus pyogenes* Cas9 CRISPR RNA (crRNA) to probe chemical and structural requirements. Ribose sugars that promoted or accommodated A-form helical architecture in and around the crRNA ‘seed’ region were tolerated best. A wider range of modifications were acceptable outside of the seed, especially *D*-2′-deoxyribose, and we exploited this property to facilitate exploration of greater chemical diversity within the seed. 2′-fluoro was the most compatible modification whereas bulkier *O*-methyl sugar modifications were less tolerated. Activity trends could be rationalized for selected crRNAs using RNP stability and DNA target binding experiments. Cas9 activity *in vitro* tolerated most chemical modifications at predicted 2′-hydroxyl contact positions, whereas editing activity in cells was much less tolerant. The biochemical principles of chemical modification identified here will guide CRISPR-Cas9 engineering and enable new or improved applications.

## INTRODUCTION

**Figure 1.**
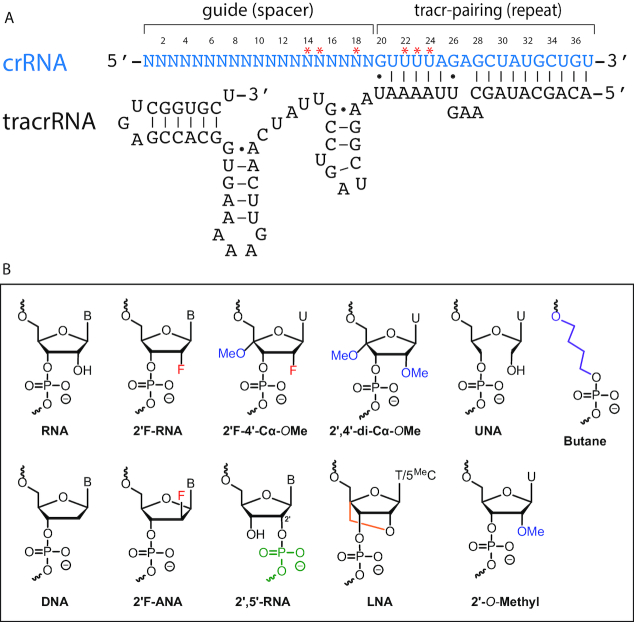
Dual RNA-guided Cas9 and sugar–phosphate chemical modifications. (**A**) Sequence and secondary structure of a dual RNA guide for *Streptococcus pyogenes* Cas9. Asterisks indicate structure-predicted 2′-OH contacts with Cas9. (**B**) Chemically modified nucleotides and linkers used in this study.

CRISPR (Clustered Regularly Interspaced Short Palindromic Repeats) is an adaptive immune defence system that evolved to combat foreign invading nucleic acids in bacteria and archaea (1–6). Their core enzymatic component typically comprises a CRISPR-associated (Cas) endonuclease bound to CRISPR RNA (crRNA) cofactors that guide sequence-specific binding and subsequent phosphodiester bond cleavage of double-stranded DNA (7,8). A well-characterized and prototypical system is CRISPR-Cas9 from *Streptococcus pyogenes* (8,9). The *S. pyogenes* crRNA is complementary to target DNA at its 5′ guide and base-pairs with a *trans*-activating crRNA (tracrRNA) via its 3′ repeat sequence. The tracrRNA uses 3′ stem-loops to bind Cas9 while the crRNA makes several direct Cas9 contacts, including six predicted 2′-hydroxyl contacts (Figure 1A) (7,10,11). The *S. pyogenes* Cas9 can also use an artificial single-guide RNA (sgRNA) where the crRNA and tracrRNA are artificially fused together (8,12,13).

CRISPR systems are being actively co-opted for biotechnology, therapeutics and synthetic biology (14,15) and further development will depend on a more detailed understanding of mechanistic principles (7,16). Chemical modification of RNA is an effective method to probe mechanism and RNA–protein relationships within ribonucleoprotein (RNP) enzymes (17). Ribose modifications can characterize the contributions of RNA to helical structure, sterics, flexibility, sugar pucker and 2′-hydroxyl requirements (18). In addition, chemical modifications can impart nuclease resistance, improved bioavailability and increased target affinity (19,20). Ribose modification has been the subject of efforts to enhance the binding, stability and function of nucleic acids in antisense oligonucleotide (AON), RNA interference (RNAi) and aptamer technologies. Chemical modifications at the 2′-position of the sugar ring have been indispensable for transitioning nucleic acids into FDA-approved drugs (21,22). It is likely that CRISPR-based technologies will also benefit from chemical modification of RNA (14,23–25).

Several studies have focused on chemical modification of crRNA, tracrRNA and sgRNA for their compatibility with gene editing applications (25–30). However, these studies primarily relied on a limited set of commercially available modifications to maintain gene editing, which involves multiple steps and poorly understood mechanisms inside of cells. The application-driven nature of these studies has made it difficult to extract guidelines based on enzyme structure or intrinsic biochemical mechanisms. For example, it has been concluded by several studies that RNA cannot be removed at certain positions, particularly the ‘seed’ region or those that make 2′-hydroxyl contacts with Cas9 (27–29,31). However, we previously found that Cas9 biochemical cleavage activity was unaffected when all of the RNA residues at these critical positions were simultaneously substituted with DNA (32). To better understand the underlying rules governing crRNA modification, we incorporated a broader and more diverse set of 2′-modified DNA and RNA analogues in crRNA and characterized their impact on Cas9’s cleavage activity. We sought to specifically address the biochemical requirement of RNA by investigating the role of A-form structure, conformational flexibility and steric constraints.

Here, we identify guidelines for modification of crRNA that are compatible with Cas9 biochemical activity. We propose that the seed region of crRNA guides prefer an A-form-like architecture whereas the tracrRNA-pairing (tracr-pairing) repeat region is compatible with a broader array of modifications, including DNA, 2′F-ANA and 2′,5′-RNA. Despite strong A-form conformational preference, relatively bulky modifications such as C2′- or C4′-*O*Me groups were only tolerated in the seed when minimal replacements were made. The RNA analogue 2′F-RNA was the most compatible modification in most positions. The high compatibility of DNA nucleotides outside of the seed region suggests that flexibility may be an important factor in conformational transitions of Cas9 (7,33,34). Chemical modification at positions of predicted Cas9–2′-hydroxyl contacts reduced efficient gene editing in cells despite efficient cleavage activity *in vitro*. These results help elucidate the functional role of RNA and provide more rational guidelines for chemical modification of CRISPR-Cas9 for synthetic biology, biotechnology and future therapeutic applications.

## MATERIALS AND METHODS

### Chemically modified oligonucleotide synthesis

Standard phosphoramidite solid-phase synthesis conditions were used for the synthesis of all modified and unmodified oligonucleotides. Syntheses were performed on an Applied Biosystems 3400 or Expedite DNA Synthesizer at a 1 micromole scale using Unylink CPG support (ChemGenes). All phosphoramidites were prepared as 0.15 M solutions in acetonitrile (ACN), except DNA, which was prepared as 0.1 M solutions. 5-Ethylthiotetrazole (0.25 M in ACN) was used to activate phosphoramidites for coupling. Detritylations were accomplished with 3% trichloroacetic acid in CH_2_Cl_2_ for 110 s. Capping of failure sequences was achieved with acetic anhydride in tetrahydrofuran (THF) and 16% N-methylimidazole in THF. Oxidation was done using 0.1M I_2_ in 1:2:10 pyridine:water:THF. Coupling times were 10–15 min for RNA, 2′F-ANA, 2′F-RNA, 2′F,4′*O*Me-RNA, 2′,4′-di*O*Me-RNA and LNA phosphoramidites. Mixed sugar modifications were prepared using premixed 1:1 equivalents of RNA 2′-amidite with RNA 3′-amidite, or 1:1 equivalents of RNA 2′-amidite or DNA 3′-amidite, or 1:1 equivalents of RNA 2′-amidite with 2′F-RNA 3′-amidite. This 1:1 ratio results in ∼0.77:1 incorporation of 2′-amidite to 3′-amidite. Deprotection and cleavage from the solid support was accomplished with either 3:1 NH_4_OH:EtOH for 48 h at room temperature (RT), or at 55°C for 16 h. Oligonucleotides containing RNA were synthesized with standard 2′-TBDMS phosphoramidites, and desilylation was achieved with either neat triethylamine trihydrofluoride for 48 h at RT, or with triethylamine trihydrofluoride/N-methyl pyrrolidone/triethylamine (1.5:0.75:1 by volume) for 2.5 h at 65°C. Crude oligonucleotides were purified by anion exchange HPLC on an Agilent 1200 Series Instrument using a Protein-Pak DEAE 5PW column (7.5 × 75 mm) at a flow rate of 1 ml/min. The gradient was 0–24% of 1 M LiClO_4_ over 30 min at 60°C. Samples were desalted on NAP-25 desalting columns according to manufacturer protocol. Modified crRNAs were prepared for RNP assembly by heating to 95°C then placing on ice to prevent formation of stable secondary structures.

### RNA and RNA–DNA oligonucleotide synthesis

DNA oligonucleotides and DNA–RNA chimeric oligonucleotides were synthesized by Integrated DNA Technologies (IDT). Chimeric crRNAs were prepared for RNP assembly by heating to 95°C then placing on ice to prevent formation of stable secondary structures. Cas9 tracrRNAs were prepared by T7 RNA polymerase *in vitro* transcription with DNA templates synthesized by IDT. Single-stranded DNA templates were annealed to T7 promoter oligo to generate double-stranded promoter regions, which support *in vitro* transcription by T7 RNA polymerase. *In vitro* transcriptions were performed by standard protocols for 2 h. Briefly, reactions contained purified T7 RNA polymerase (4 μM), 30 mM Tris (at pH 7.9), 12.5 mM NaCl, 40 mM MgCl2, 2% PEG 8000, 0.05% Triton X-100, 2 mM spermidine and 2.5 μM T7-DNA template. Afterward, the DNA template was degraded by DNase I treatment. Reactions were phenol–chloroform extracted and gel-purified from denaturing polyacrylamide gels. Purified RNA was refolded by heating to 95°C for 5 min in a heating block followed by slow cooling the block to RT (∼40 min). RNA was quantified by measuring absorbance at 260 nm and calculated extinction coefficients using nearest neighbor approximations and Beer’s Law.

### Preparation of Cas9 enzymes

Plasmid encoding an *Sp*Cas9 (simply referred to as Cas9) with a C-terminal fusion of a nuclear localization signal (NLS) and a 6x-Histidine tag (pET-Cas9-NLS-6xHis) was obtained from Addgene (62933). A dead Cas9 (dCas9) version was prepared by performing site-directed mutagenesis on this plasmid to generate H840A and D10A mutations (pET-dCas9-NLS-6xHis). Cas9 proteins were prepared similar to that previously described (35). Briefly, protein expression was induced in Rosetta (DE3) cells with 0.4 mM isopropyl β-D-1-thiogalactopyranoside (IPTG) at 18°C for 16 h. Cell pellets were resuspended in 12 ml of chilled binding buffer (20 mM Tris–HCl, pH 8.0, 250 mM NaCl, 1 mM PMSF, 5 mM imidazole) per 0.5 l of culture pellet. Resuspended cells were sonicated and clarified by centrifugation. Supernatant was purified by His-Pur Cobalt-CMA resin (Thermo Scientific) by sequentially increasing concentrations of NaCl wash buffer (Tris–HCl, pH 8, 0.25/0.5/0.75/1.0 M NaCl, 10 mM imidazole). Protein was eluted with 130 mM imidazole wash buffer. The eluent was concentrated and exchanged into 2x Cas9 storage buffer (40 mM HEPES-KOH, pH 7.5, 300 mM KCl, 2 mM ethylenediaminetetraacetic acid (EDTA), 2 mM DTT) then one volume of glycerol added. Concentration was determined by UV absorbance at 280 nm using a calculated extinction coefficient (120 450 M^−1^ cm^−1^) and Beer’s law.

### *In vitro* Cas9 cleavage activity assays

*In vitro* cleavage assays were performed as previously described (32). Linearized plasmid target DNA (100 ng) harboring the tetracycline receptor (TR) gene or EGFP (EG) were combined with the Cas9 RNP complex (0.75 μM Cas9, 0.25 μM tracrRNA, 0.3 μM crRNA final concentration) in a 1× cleavage buffer (20 mM Tris–HCl, pH 7.5, 100 mM KCl, 5% glycerol, 1 mM DTT, 0.5 mM EDTA, 2 mM MgCl_2_) supplemented with 0.1 mg/ml of purified yeast tRNA. The concentration of tracrRNA was purposely set as the limiting component of the RNP complex and used to predict final RNP concentration. Molar excess of Cas9 and crRNA will ensure complete assembly of tracrRNA into RNP complexes and does not result in aggregation at concentrations used in our assays (32). Standard reaction conditions were 37°C for 2 h in a final reaction volume of 40 μl. The mixture was treated with 10 μg of RNase A (Thermo Scientific) for 15 min followed by 20 μg of Proteinase K (Thermo Scientific) for 15 min at room temperature. The DNA products were precipitated in 10 volumes of acetone with 2% LiClO_4_ at −20°C for >1 h. DNA cleavage products were resolved by agarose electrophoresis and visualized using ethidium bromide staining. The fraction of target plasmid cleaved was quantified using ImageJ software. The band intensity for the cleavage product band was divided by the combined intensity of the largest cleavage product and uncut substrate plasmid bands and reported as fraction cleaved (i.e. ‘cut’/‘cut + uncut’). Error bars for all quantified data represent experimental replicates, not technical replicates. Sample size was selected based on the expectation that three or more replicates will be representative of typical *in vitro* assay conditions.

### Radiolabeling of DNA target

A total of 100 pmols of antisense DNA target strand was radiolabeled with [γ- 32P]-ATP using T4 polynucleotide kinase following the manufacturer’s recommended enzyme protocol (Thermo Fisher). Reactions were phenol-chloroform extracted and radiolabeled DNA was gel-purified on 15% denaturing polyacrylamide gels by the crush-and-soak method. Gel-purified radiolabeled RNA and DNA was quantified by scintillation counting.

### Dot-blot filter binding assays for duplex target binding

For target binding by Cas9 RNP complexes, radiolabeled duplex target DNA (1000 cpm/reaction) was combined with increasing concentrations of a pre-assembled dCas9–tracrRNA–crRNA complex in a final reaction of 20 μl 1× cleavage buffer and 0.1 mg/ml tRNA. After incubation at 37°C for 15 min, reactions were vacuum filtered over nitrocellulose membrane (Protran Premium NC, Amersham) using a 96-well dot blot apparatus. Wells were washed twice with 200 μl of 1× cleavage buffer. Membrane was then removed and washed with 1× PBS solution three times and air dried at RT. Binding of radiolabeled DNA was then visualized by GE Typhoon phosphorimager. Spots were quantified with ImageQuant software, values plotted in Prism (GraphPad) and data fit to a one-site binding hyperbola equation.

### Thermal denaturation monitored by UV absorbance

Thermal denaturation was as previously described (32). Cas9 alone or complexed to tracrRNA and crRNA at 1 μM final concentration (equimolar concentrations of all components) was incubated at room temperature for 10 min in degassed 1× UV melt buffer (20 mM Cacodylate, pH 7.5, 150 mM KCl, 1 mM MgCl_2_). Samples were melted in a Cary 400 UV/Vis spectrophotometer at a ramp rate of 1°C/min while UV absorbance at 280 nm was collected every 1 min. Experiments were repeated in duplicate. Melting temperatures were determined using Van’t Hoff calculations and error determined by standard error of the mean using two experimental replicates for each sample. Melt data was plotted using Prism (GraphPad) software.

### Cell-based editing measured by flow cytometry

HEK 293T cells expressing EGFP and *Sp*Cas9 were a kind gift from Wen Xue (UMass Medical Center) (29). Cells were grown in Dulbecco’s modified eagle’s medium (DMEM) with 1× non-essential amino acids (NEAA), 5% Cosmic calf serum (CCS) and 2.5% fetal bovine serum (FBS) without antibiotics. Cells were reverse transfected (40–50 000 cells) in four experimental replicates in 96-well plates with 20 pmols of crRNA:tracrRNA complex in a final of 200 μl using RNAiMAX (Invitrogen) following the manufacturer’s recommended protocol. After 12 h, one volume of media containing 15% FBS and 1× Penicillin-Streptomycin solution was added to the Opti-MEM and cells incubated for an additional 12 h. Media was then replaced with full media and cells grown for an additional 4 days.

For flow cytometry, cells were washed with PBS, trypsinized, washed again and then fixed with 1% paraformaldehyde in PBS for 8 min. Cells were washed again and counted in an Accuri C6 Flow Cytometer. EGFP was detected using the blue laser at excitation 488 nm; emission detection 530 ± 15 nm (FL1 channel). At least 20 000 events were collected and analyzed by Accuri CFlow Plus software. The cells were first gated based on forward and side scattering (FSC-A/SSC-A) to remove cell debris, then gated to select single cells (FSC-H/FSC-A). At last, cells were gated to select EGFP positive cells. The quadrant gate was established using the signal from non-EGFP expressing control cells. Untreated HEK 293T cells expressing EGFP and Cas9 contained ∼6% non-fluorescent cells. The average from four replicates was used for background subtraction to determine the extent of cell-based editing after treatment.

## RESULTS

We used a dual RNA-guided (separate crRNA and tracrRNA) *S. pyogenes* CRISPR-Cas9 to probe the role of the guide RNA in enzyme activity (Figure 1A). As opposed to an artificial single-guide RNA (sgRNA) (8), dual RNA-guided Cas9 reflects the naturally occurring enzyme (36,37). Given their shorter length, dual guides are also more amenable to chemical modification (28). To facilitate more efficient crRNA chemical synthesis, we truncated a crRNA by 2 nt at the 5′ end and 4 nt at the 3′ end to yield a 36 nt crRNA (28). For this study, we have selected several modified nucleotides with properties that mimic or antagonize the properties of RNA (Figure 1B). To isolate and compare the effects of these modifications, we initially used a guide sequence targeting the tetracycline receptor (TR) gene and quantified site-specific *in vitro* cleavage of a linearized plasmid (Supplementary Figure S1) (32).

### Complete crRNA modification can support target DNA cleavage activity

**Figure 2.**
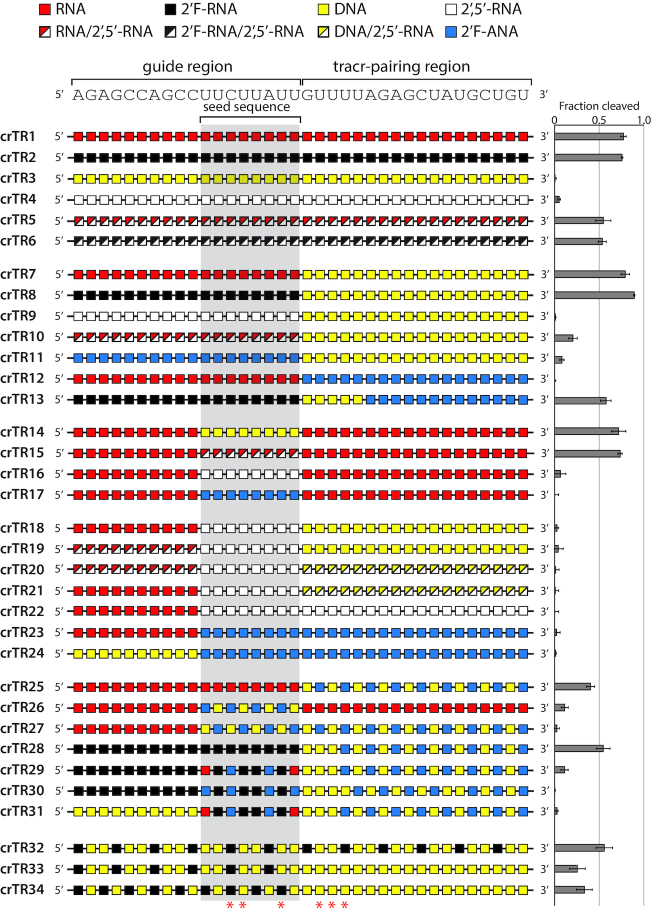
Modification schemes and the corresponding Cas9 enzyme activity for chemically modified crRNAs. Entire crRNAs or large sequence tracts in the guide, seed or tracrRNA-pairing region are modified. crRNA sequence is shown above and structure-predicted 2′-OH contacts with Cas9 are indicated with asterisks below. Enzyme activity is reported to the right. Error is reported as standard error of the mean (s.e.m.) for three or more replicates.

To establish a baseline for modification effects, we completely modified a native crRNA (crTR1) to 2′F-RNA (crTR2), a modification that strongly mimics RNA sugar pucker but cannot donate hydrogen bonds (38) (Figure 2). Enzyme activity was very high, comparable to crTR1, suggesting that strong A-form conformation and small chemical changes are preferred whereas hydrogen bonding may be less important. We also synthesized a control crRNA composed entirely of DNA (crTR3). Consistent with our earlier report, crTR3 did not support target DNA cleavage (32). A DNA–DNA hybrid with the target would adopt a B-form helical structure (39) not easily accommodated by Cas9 (10,11).

2′,5′-RNA is an RNA mimic where 3′,5′-phosphodiester linkages are replaced by the regioisomeric 2′,5′ linkage; it binds to complementary native RNA with good affinity, and incorporation of a few 2′,5′ linkages into an otherwise unmodified RNA strand has a minor impact on duplex stability (40). The 2′,5′-RNA modification is known to induce ‘A-like’ structure on oligonucleotides and sequences containing this modification prefer to hybridize to RNA over single-stranded DNA (ssDNA) (41,42). Thus, 2′,5′ linkages should serve as an alternative A-form structure probe. Complete conversion of a crRNA to 2′,5′-RNA abrogated enzyme activity (crTR4), possibly reflecting its poor affinity towards single-stranded DNA. To mitigate overpowering effects of 2′,5′ modifications at individual positions and generate a more moderate probe, we incorporated a mixture of 2′,5′ and 3′,5′ linkages at each position. Recent high-resolution crystallographic data on mixed-backbone RNA duplexes show that RNA duplexes containing a few 2′,5′ linkages share the same global A-like structure as the native duplex (41,43). Remarkably, RNA helical structures are well retained with 40% backbone heterogeneity (43). The result is a mixed population of crRNA regioisomers with the same base sequence. The resulting crRNA crTR5 consisted of a statistical mixture of 2^35^ (or 3.44 × 10^10^) isomers. A similar isomeric effect is observed with phosphorothioate linkages, which has not been a major hurdle for their use in development of FDA-approved drugs like Nusinersen (44). Incorporating 3′,5′ linkages into the inactive 2′,5′-crRNA strand significantly restored activity (crTR4 versus crTR5), albeit not to the level of a native 3′,5′ crRNA (crTR1). Combining 2′,5′-RNA with 2′F-RNA in a mixed configuration (crTR6) also provided substantial activity, also supporting the A-form-like structural accommodation of 2′,5′ linkages.

We and others have previously found that Cas9 enzymes can accept partial RNA–DNA chimeric crRNAs (29,31,32). In particular, converting the entire tracr-pairing region to DNA significantly enhanced cleavage activity and maintained or enhanced specificity (32). Thus, we rationalized that synergy might be achieved by taking into consideration the modular nature of the crRNA. We modified the guide and tracr-pairing regions independently and confirmed that DNA in the tracr-pairing region was highly compatible with our target sequence (crTR7) (32). DNA in this region will pair with RNA in the tracrRNA and likely maintain an A-form architecture while increasing conformational freedom (32). While keeping DNA in the tracr-pairing region, we then substituted 2′F-RNA (crTR8), 2′,5′-RNA (crTR9), RNA/2′,5′-RNA mixed nucleotide modification (crTR10) and 2′F-ANA (crTR11) into the guide region. The 2′F-RNA configuration was very compatible, supporting high activity. The 2′,5′-RNA and RNA/2′,5′-RNA mixed nucleotides exhibited little or no activity.

### The guide/seed region strongly prefers A-form helical structure

2′F-ANA (2′-deoxy-2′-fluoro-arabinonucleic acid), the 2′ epimer of 2′F-RNA, is known to acquire a C2′/*O*4′-*endo* sugar pucker and prefer a B-form helix (45,46), which results in more DNA-like properties but with less flexibility. The 2′F-ANA configuration (crTR11) was inactive. These results suggested that the guide region is more reliant on A-form helical structure than the tracr-pairing region. This is not unexpected since it will pair with a DNA target and must assume an A-form-like conformation for efficient catalysis (10,11). We therefore tested B-form conformational preference in the tracr-pairing region by substituting in 2′F-ANA (crTR12). This modification configuration was also completely inactive. Since 2′F-ANA lacks the conformational flexibility of DNA (45), it is likely that 2′F-ANA in certain tracr-pairing positions is incompatible due to inflexibility. In support of this possibility, we found that a crRNA with a 2′F-RNA guide and a 2′F-ANA tracr-pairing region could support high cleavage activity when the first 4 nt of the tracr-pairing region were DNA residues (crTR13).

Within the crRNA guide region is a ‘seed’ sequence where crRNA pairing to a target DNA is nucleated (7). Previous investigations reported that seed sequence residues cannot be fully modified and specific positions require RNA (27–29,31). Likewise, crystal structures have predicted crRNA 2′-hydroxyl contacts with Cas9 (10,11). Arguing against a strict requirement for 2′-hydroxyl contacts, we previously showed that all predicted contacts could be simultaneously converted to DNA in an otherwise native crRNA with no effect on biochemical activity (32). To probe the seed sequence, we substituted eight contiguous seed residues while maintaining RNA in the rest of the crRNA. DNA (crTR14), as well as a mixed RNA/2′,5′-RNA strand (crTR15), provided high activity, indicating that predicted 2′ polar contacts are of limited importance. However, complete conversion of the seed to 2′,5′-RNA (crTR16) or 2′F-ANA (crTR17) resulted in little or no activity. To determine the importance of flanking nucleotide conformation on the seed region, we started with 2′,5′-RNA or 2′F-ANA in the seed and then substituted flanking regions with various modifications (crTR18-crTR24). None of these combinations supported activity, suggesting an inability to strongly influence seed structure. Therefore, the seed region appears to strongly prefer A-form helical structure and is not significantly impacted by flanking modifications. An exception may be observed for conformationally flexible nucleotides like DNA (crTR14).

Based on the above results, we tested a variety of ‘altimers’ where two or more modifications are alternated in a pattern (47). Altimers can exploit unique synergy between adjacent modifications and modulate effects. Indeed, altimers of DNA and 2′F-ANA in the tracr-pairing region supported high activity when coupled with RNA (crTR25) or 2′F-RNA (crTR28) in the guide region. The seed region was not compatible with altimer configurations using 2′F-ANA (crTR29-crTR31). However, altimers of 2′F-RNA and DNA across the seed region were generally tolerated (crTR32-crTR34), further supporting a predominant role for A-form conformational effects and the benefit of conformational flexibility (32).

**Figure 3.**
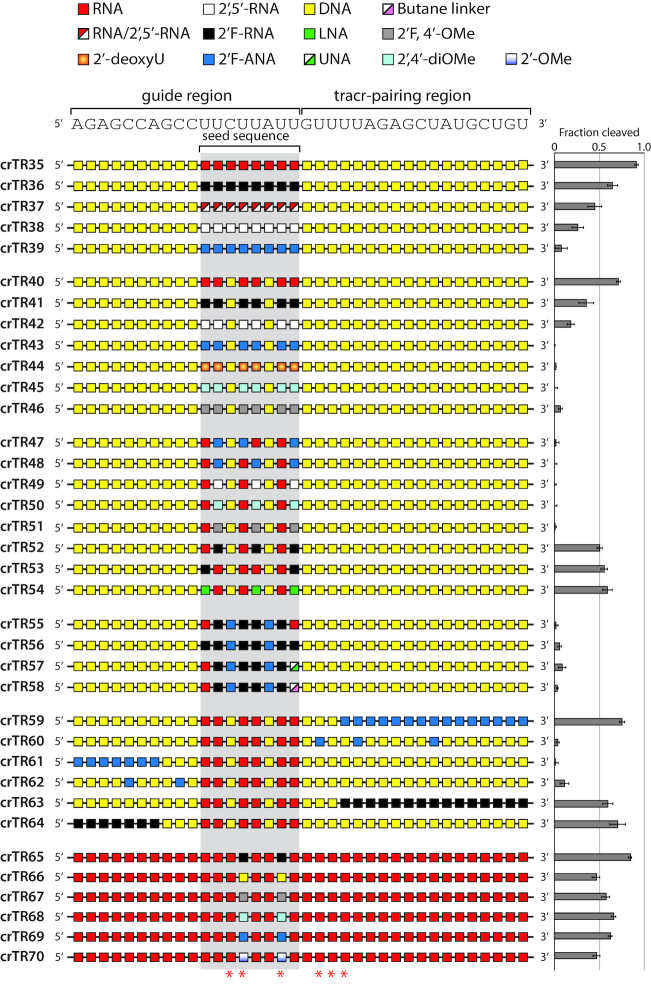
Modification schemes investigating effects on Cas9 enzyme activity primarily at the seed region. crRNA sequence is shown above and structure-predicted 2′-OH contacts with Cas9 are indicated with asterisks below. Enzyme activity is reported to the right. Error is reported as s.e.m for three or more replicates.

Our previous study characterizing RNA–DNA chimeric crRNAs found that RNA residues in the seed region alone, with DNA in all other positions, was sufficient to support robust *in vitro* enzyme activity by Cas9 (32). We confirmed this effect (crTR35) and used the high compatibility of DNA outside of the seed region to further probe the structural and conformational requirements of the seed region (Figure 3). In this configuration, there is a lack of strong interference from flanking sequence because of the relative conformational neutrality of DNA. Once again, we found that 2′F-RNA was a suitable replacement for RNA (crTR36). As well, 2′,5′-RNA (crTR38) or 2′-FANA (crTR39) in the seed region showed little activity. Mixed linkages (2′,5′/3′,5′-RNA) in the seed were more efficient (crTR37) than 2′,5′-RNA, reinforcing our earlier observation that RNA can help mitigate the structural effects of 2′,5′-linkages.

We wanted to explore more diverse modifications, some of which are only available as uridines. Therefore, we modified the six uridines in the 8-nt seed region while keeping all other positions DNA. With only six uridine nucleotides to modify, we explored replacement of RNA (crTR40) with 2′F-RNA (crTR41), 2′,5′-RNA (crTR42), 2′F-ANA (crTR43), DNA (crTR44) (to distinguish thymine versus uracil effects), 2′,4′-di-Cα-*O*Me RNA (crTR45) and 2′F-4′-Cα-*O*Me RNA (crTR46) (48–51). Other than 2′F-RNA, and to a small degree 2′,5′-RNA, none of these modifications supported activity. This was surprising given the RNA-like (C3′-*endo*) sugar pucker previously reported for 2′,4′-di-Cα-*O*Me and 2′F-4′-Cα-*O*Me (48,50,51). Multiple 4′-*O*-methyl groups may introduce unfavorable steric constraints due to their bulkiness relative to a hydrogen atom.

Nearest neighbor effects can also modulate the conformation or properties of adjacent nucleotides. For example, DNA is easily induced into A-form-like conformation by neighboring RNA nucleotides (52,53). Likewise, juxtaposing 2′F-ANA and 2′F-RNA next to each other can relax the strong RNA-like properties of 2′F-RNA (54). To determine whether nearest neighbor effects could make modifications work synergistically, we combined RNA with 2′F-ANA in the seed (crTR47 and crTR48). RNA was unable to improve activity for 2′F-ANA in the seed. Combining RNA with 2′,5′-RNA (crTR49), 2′,4′-di-Cα-*O*Me (crTR50), or 2′F-4′-Cα-*O*Me (crTR51) also resulted in no activity. Thus, RNA was unable to offset the unfavorable effects of these modifications. In contrast, combining RNA with 2′F-RNA (crTR52 and crTR53) and LNA (crTR54), a 2′-4′ bridging modification that locks C3′-*endo* sugar pucker (55), resulted in robust enzyme activity. Combining 2′F-RNA (with and without RNA) with 2′F-ANA was unable to restore activity (crTR55 and crTR56). Attempting to increase conformational freedom by placing a UNA nucleotide (56) (crTR57) or butane linker (crTR58) at the very 3′ nucleotide of the seed region did not restore activity.

2′F-ANA was well tolerated in the tracr-pairing region, as long as a few of the first 5′ nucleotides were DNA (crTR13 and crTR59). However, placement of 2′F-ANA at the second position (along with the fifth and eleventh positions) in the tracr-pairing region (crTR60) abrogated activity. Combined with the activity of 2′F-ANA-containing crRNAs crTR12, crTR13, crTR25, crTR27 and crTR28, this result suggests that one or more positions closest to the seed must assume A-form structure, either through conformational predisposition or flexibility. DNA may temper the formation of A/B junctions or rigid versus flexible A-form transitions between the seed and tracr-pairing region. Placement of 2′F-ANA outside of the seed but still in the guide region (crTR61 and crTR62) was also detrimental to activity. Conversely, placing 2′F-RNA in the same guide and tracr-pairing positions resulted in robust activity (crTR63 and crTR64). These results reinforce a strong preference for A-form helical structure in the guide region, especially the seed sequence and a greater tolerance for modification in most of the tracr-pairing region. This difference may derive from hybridization of the crRNA 5′ guide region to a DNA target versus pairing of the 3′ region to a tracrRNA partner.

To further probe the role of RNA in the seed region, we replaced two uridines that are predicted to mediate 2′-hydroxyl contacts (10,11) while maintaining RNA in all other positions (crTR65-crTR70). Replacement with 2′F-RNA was the most compatible (crTR65), while DNA (crTR66), 2′F-4′-Cα-*O*Me (crTR67), 2′,4′-di-Cα-*O*Me (crTR68) and even 2′F-ANA (crTR69) were well-tolerated. The incorporation of 2′-*O*-methyl, a known RNA conformational mimic (57), was only moderately effective, reinforcing steric issues with bulky modifications at the 2′ position. We selected only uridines for replacement, so sequence or base composition may influence modification effects. However, these results show that judicious placement in a few seed positions can make modifications tolerable, even in sensitive 2′-hydroxyl contact positions.

### RNP stability, target DNA engagement and a second guide sequence support crRNA activity trends

**Figure 4.**
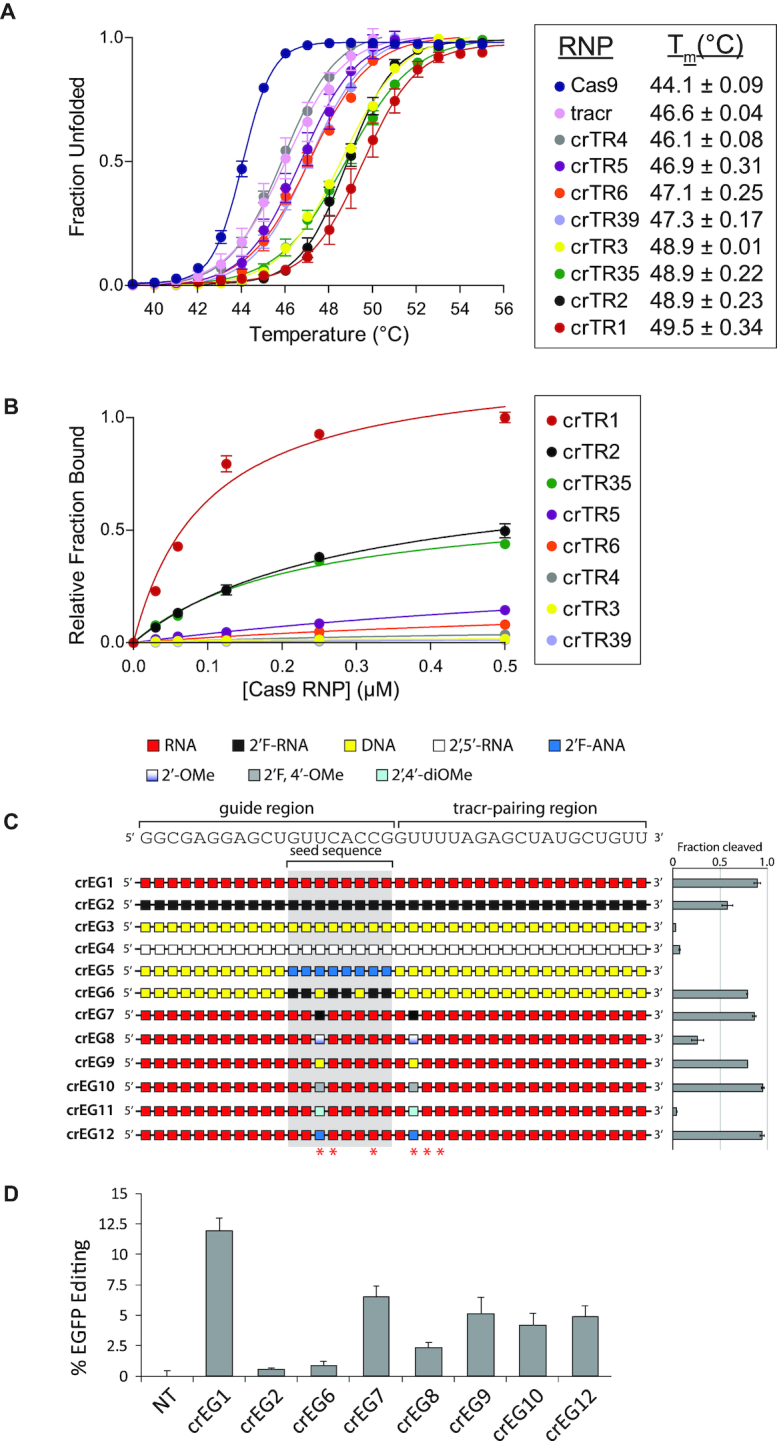
Thermal denaturation, target binding and a second guide sequence help rationalize crRNA activity trends. (**A**) RNP thermal denaturation monitored by UV absorbance at 280 nm. Error is reported as s.e.m. for two replicates. (**B**) Substrate engagement measured by dot-blot filter binding of radiolabeled target DNA. Error is reported as s.e.m. for three or more replicates. (**C**) *In vitro* cleavage activity of Cas9 RNPs assembled with chemically modified crRNAs targeting an EGFP gene. crRNA sequence is shown above and structure-predicted 2′-OH contacts with Cas9 are indicated with asterisks below. Enzyme activity is reported to the right. Error is reported as s.e.m. for three or more replicates. (**D**) Cell-based editing activity of crRNAs co-transfected with tracrRNA into HEK 293T cells stably expressing EGFP and Cas9. Gene editing efficiency was measured as loss of EGFP fluorescence by flow cytometry 5 days post-transfection. Error is reported as s.e.m. for four replicate treatments.

To rationalize the effect of modifications, we assembled Cas9 RNP complexes and determined their thermal stability (Figure 4A) and their binding to a double-stranded DNA target (Supplementary Figure S2 and Figure 4B). Thermal stability was measured by absorbance at 280 nm as temperature was increased (32). Cas9 alone exhibited a melting temperature (*T*_m_) of 44°C. Upon assembly with a tracrRNA, the *T*_m_ increased to 46.6°C. Higher order assembly with native crRNA (crTR1) resulted in an additional increase in the *T*_m_, up to 49.5°C. We selected a handful of representative crRNAs to determine their impact on RNP stability. Two crRNAs that provided high enzyme activity, crTR2 and crTR35, fell into a high *T*_m_ regime like native crRNA. One exception was crTR3, composed entirely of DNA. This modified oligonucleotide seems to assemble stable RNP complexes but does not support catalysis (32). crRNAs that fell into an intermediate *T*_m_ regime generally supported moderate activity (crTR5 and crTR6). However, crTR39, with 2′F-ANA in the seed, also fell into the intermediate *T*_m_ range. This result and the *T*_m_ of crTR3 illustrate the modular nature of crRNAs where tracrRNA-pairing might support assembly, yet the guide cannot support catalysis. Only crTR4, composed entirely of 2′-5′ linkages, did not increase RNP *T*_m_. This crRNA may be unable to efficiently assemble a stable RNP complex with Cas9-tracrRNA, explaining its lack of activity.

The ability of these chemically modified crRNAs to stably engage target DNA mirrored the observations from RNP stability (Figure 4B and Supplementary Figure S2). Native crRNA (crTR1) facilitated strong equilibrium binding of Cas9 RNP to target double-stranded DNA. Two crRNAs with high activity and stable RNP assembly, crTR2 and crTR35, exhibited moderate but stable target binding. crRNAs with reduced enzyme activity (crTR3 and crTR4) had lower target binding and those that possessed no cleavage activity did not bind the target. The all-DNA crTR3, while able to assemble a stable RNP complex, was unable to engage target DNA, as previously reported (32). Thus, the effects of chemical modification can be correlated with RNP assembly and target substrate engagement.

To determine if some of the general principles we have observed could be extended to another sequence, we selected a few modification schemes and a new guide sequence that targets an EGFP gene. We performed *in vitro* cleavage assays as before but using a linearized plasmid harboring the EGFP gene as substrate (Figure 4C). A native crRNA targeting EGFP (crEG1) supported about 90% cleavage of the target plasmid. Complete modification with 2′F-RNA (crEG2) resulted in substantial, albeit reduced, cleavage at about 55%. As expected, complete conversion to DNA (crEG3) or 2′,5′-RNA (crEG4) resulted in loss of activity. For the crTR guide we showed that DNA outside of the seed region was compatible with cleavage activity if the seed contained modified nucleotides with appropriate RNA-like properties. This was confirmed by testing either 2′F-ANA or 2′F-RNA in the seed. The 2′F-ANA seed did not support enzyme activity (crEG5) whereas 2′F-RNA in only six positions in the seed region was well-tolerated (crEG6). These results support the overall preference for A-form like structure, especially in the seed region and the compatibility of DNA in other regions of the crRNA.

To extend some of these results to Cas9 activity inside of cells, we co-transfected tracrRNA and crRNA modified with 2′F-RNA (crEG2) or 2′F-RNA and DNA (crEG6) into HEK 293T cells stably expressing EGFP and Cas9. These modified guides target EGFP and should result in loss of EGFP expression if gene editing was successful. Editing was measured by flow cytometry five days after transfection. Despite high activity *in vitro* and the presence of the strong RNA mimic 2′F-RNA, these guides failed to produce substantial editing activity (Figure 4D). A recent report suggested that modification at certain predicted 2′-hydroxyl contacts is a bottleneck for complete modification of editing-active crRNAs (27).

To focus on guides that might reveal the impact of chemical modifications at these positions, we generated a new set of modified crRNAs (crEG7-crEG12) against the same EGFP sequence. In this case, however, only two predicted 2′-hydroxyl contact positions were modified. These modified crRNAs are similar to a subset tested *in vitro* in Figure 3 (crTR65-crTR70), but the modifications were placed at two different positions. The positions chosen contained uridine nucleotides to allow testing of a more diverse variety of chemical modifications. These two positions were also shown to be sensitive to modification in a previous report (27).

*In vitro* cleavage revealed that these crRNAs either supported high activity (crEG7, 9, 10 and 12), moderate activity (crEG8), or no activity (crEG11) (Figure 4C). All except crEG11 were subsequently co-transfected with tracrRNA and their ability to support editing was measured by flow cytometry. These crRNAs showed editing efficiencies that were lower than an unmodified crRNA, but quite similar to the trends observed *in vitro* (Figure 4D). These results underscore the importance of predicted 2′-hydroxyl contacts with Cas9 for maintaining high editing efficiency in cells. They also support our *in vitro* observations regarding the general compatibility of chemical modifications that prefer or can assume A-form helical structure and are not sterically bulky.

## DISCUSSION

The potential use of CRISPR-Cas9 for gene editing in patients, and its possible other applications in pharmacotherapy, will likely face key hurdles not unlike other oligonucleotide-based therapeutics (19–22). Very early work with AONs and siRNAs was undertaken with unmodified, natural molecules. However, it soon became clear that native oligonucleotides were subject to relatively rapid degradation and lacked sufficient drug-like properties, such as biodistribution or cellular uptake (19,58). Although CRISPR-Cas9 is a large RNP enzyme that possesses unique challenges for drug development (14,23,24), it is anticipated that chemical modification of the guide RNA will significantly benefit certain therapeutic applications (59). Previous investigations into CRISPR-Cas9 RNA chemical modification have generally focused on screens for guide RNAs that are tolerated in gene editing applications with an emphasis on nuclease resistance or off-target effects. These have included modification of sgRNA with 2′-*O*-methyl, 2′F-RNA, and phosphorothioate (PS) or thiophosphonoacetate linkages at the termini (26) or internal residues (30), partial substitution of crRNAs with DNA (29) or 2′F-RNA, 2′-*O*-methyl, 2′-4′ bridged nucleic acid and PS linkages (28), or site-specific incorporation of 2′-*O*-methyl-3′-phosphonoacetate (25) and 2′-4′ bridged nucleic acids (60). A recent study explored extensive structure-guided modification with 2′F-RNA, 2′-*O*-methyl and PS linkages in both the crRNA and tracrRNA and found that heavy modification could still support satisfactory gene editing (27). While informative, these studies did not fully establish clear mechanistic or rational rules for guide RNA modification or correlated gene editing results with a biochemical understanding of Cas9 RNP structure–function.

In this study, we sought to establish more fundamental rules for chemical modification that correlate with the intrinsic biochemical activity of Cas9. These guidelines are likely to impact a wide range of CRISPR applications. In a previous study of crRNA structure-function, we took advantage of the excellent probing properties of 2′-deoxynucleotides. Substituting RNA with DNA at specific residues provided characterization of 2′-hydroxyl contacts, steric flexibility and A-form helical structure. Systematic substitution suggested strong preferences for A-form helical structure in the crRNA, a lack of requirement for 2′-hydroxyl contacts with Cas9, and potential regulatory features of the crRNA that could enhance Cas9 activity (32). Here we more rigorously tested our hypothesis of A-form helical structure requirements for the crRNA while evaluating the impact of conformational flexibility and steric restrictions on Cas9 activity.

We used both commercially available and custom modified nucleotides that can mimic RNA or DNA nucleotides and better control crRNA conformation, flexibility, polar contacts and steric constraints. Among the chemically modified nucleotides we tested, those possessing conformational properties similar to RNA were 2′F-RNA, 2′,5′-RNA (31–33), LNA (44), 2′,4′-di-Cα-*O*Me RNA (51), 2′F-4′-Cα-*O*Me RNA (39,40) and 2′-*O*-methyl (57). Among these, 2′F-RNA is one of the most prominent RNA substitutions in nucleic acid-based applications because of its C3′-*endo* sugar pucker that increases binding affinity to target RNA, increased endonuclease resistance, and small substituent size (38,61,62). In contrast, 2′F-ANA mimics the DNA conformation, has minimal steric constraints, possesses hydrogen bond acceptor potential and has substantial nuclease resistance (63). We found that replacing RNA bases with 2′F-RNA generally maintained an excellent degree of activity. DNA could sometimes be partially replaced with 2′F-ANA, such as in the tracr-pairing repeat region. In contrast, placing 2′F-ANA in positions that require A-form helical architecture, specifically the seed region, severely reduced activity presumably due to its inflexibility. The 2′,5′-RNA nucleotides were largely detrimental when replacing RNA. However, when 2′,5′-RNA was used to replace RNA or 2′F-RNA nucleotides in a mixed nucleotide configuration (crTR5 and crTR6), substantial cleavage was often observed. Approximately 40% activity was retained when three of six RNA bases in the seed (in an otherwise all-DNA crRNA) were replaced with LNA bases (crTR54). Replacing too many RNA bases with ribonucleotides possessing 2′F,4′-*O*Me and 2′,4′-di-*O*Me moieties resulted in complete loss of activity. Instead, these modifications performed better when placed at only one or two select positions, as in the case of siRNAs (48). The effectiveness of DNA and 2′F-RNA suggests that guide flexibility and conformational preference is a key factor in Cas9′s ability to achieve catalytically competent conformational states.

Together, our results support a need for A-form structure throughout the crRNA. A requirement in the guide region, specifically the seed sequence, likely arises from its role in nucleation of target hybridization and the sensing of specific duplex shape by Cas9 in this region (64–66). The tracr-pairing repeat region is likely to tolerate broader modification because hybridization with tracrRNA should help induce an A-form duplex architecture. The tracr-pairing region also plays a poorly understood role in modulating Cas9 activity (32). A-form conformation and flexibility at or near the junction between the guide and tracr-pairing region may offer insight into this regulation. For example, DNA or 2′F-RNA are tolerated at or near this junction, but not 2′F-ANA or 2′,5′-RNA. Our results indicate that modified nucleotides in the crRNA guide region, especially the seed sequence, that mimic the properties of RNA will be the most successful. Overuse of modifications with bulky moieties should be avoided in the seed region. The use of multiple modification types is supported, which can take advantage of synergistic effects among modifications. DNA nucleotides, for example, can offset conformationally rigid modifications like 2′F-ANA, although both offer potentially advantageous properties when judiciously placed in the tracr-pairing region.

Previous reports have suggested a difficulty in replacing predicted Cas9–2′-hydroxyl contacts with chemically modified nucleotides when trying to preserve gene editing activity in cells (27,28,31). Our investigations support this phenomenon. Although two crRNAs lacking any RNA residues (crEG2 and crEG6) performed very well *in vitro*, they supported very little gene editing inside of cells. Specifically focusing chemical modification on two 2′-hydroxyl contact positions in the crRNA (crEG7-crEG12) revealed a clear reduction in editing efficiency for all modification types tested. Thus, our results further emphasize a disconnect between the intrinsic biochemical activity of Cas9 and its ability to induce gene editing in cells that centers on a handful of 2′-hydroxyl contacts with the guide RNA. While several mechanistic causes are possible, the difference in substrate complexity between *in vitro* (plasmid DNA) and cell-based editing (chromatinized DNA) experiments is evident. Characterizing the source of discrepancy between *in vitro* and cell-based activity should provide a path forward for the generation of CRISPR-Cas9 enzymes that can utilize completely modified guides with high efficiency for gene editing applications in the future.

## CONCLUSION

This study establishes A-form helical structure and steric constraint as the primary determinants of the crRNA structure–function relationship with Cas9. Based on this observation, we identify rational guidelines for chemical modification of the crRNA sugar–phosphate backbone that are compatible with Cas9 enzyme activity. In summary, the guide region prefers an A-form-like architecture, primarily due to nucleation of target DNA pairing at the seed. The tracr-pairing region is compatible with a broader array of modifications due to hybridization with tracrRNA and possible roles in conformational regulation of Cas9 activity. Potentially bulky modifications should be avoided in the seed sequence while a broad variety of modifications are likely to be well-tolerated at crRNA 5′ and 3′ termini (26). The combination of multiple modification types can provide flexibility in tuning Cas9 activity by combining synergistic properties. The phenomenon of apparent 2′-hydroxyl requirement at certain guide positions, which are dispensable *in vitro*, pose restrictions to highly efficient gene editing by fully modified guides (27–29,31,32). The discovery of a clear mechanistic underpinning or the identification of modifications that can compensate for the positional 2′-hydroxyl requirement should unlock this restriction and make the guidelines established here highly relevant for rational guide modification.

## Supplementary Material

Supplementary Data
