# Supplementary material for: Extensive CRISPR RNA modification reveals chemical compatibility and structure-activity relationships for Cas9 biochemical activity
Source: Nucleic Acids Res. 2018 Dec 4;47(2):546–58. doi: 10.1093/nar/gky1214 (PMC6344873; doi:10.1093/nar/gky1214)
Supplement: Supplementary Data [file gky1214_supplemental_files.pdf]

Supplemental Information

Extensive CRISPR RNA Modification Reveals Chemical Compatibility and Structure-Activity Relationships for Cas9 Biochemical Activity

Daniel O'Reilly[1†], Zachary J. Kartje[2†], Eman A. Ageely[2], Elise Malek-Adamian[1], Maryam Habibian[1], Annabelle Schofield[1], Christopher L. Barkau[3], Kushal J. Rohilla[3], Lauren B. DeRossett[2], Austin T. Weigle[2], Masad J. Damha[1,*], and Keith T. Gagnon[2,3,*]

[1]Department of Chemistry, McGill University
[2]Department of Chemistry & Biochemistry, Southern Illinois University
[3]Department of Biochemistry & Molecular Biology, School of Medicine, Southern Illinois University

[†]Equally contributing Authors
*Corresponding Authors

Table 1. Synthesized crRNA with predicted masses and mass found by HRMS.

Sample Number	Sample Name	Predicted mass	Observed Mass	Sample Number	Sample Name	Predicted mass	Observed Mass
**cr1**	36RNACon	11426.71	11426.85	**cr37**	crTR_S_2'-5'-l	11091.23	11089.61
**cr2**	FRA	11498.78	11497.44	**cr38**	crTR FANAk	11191.04	11191.91
**cr3**	mix3	11498.78-11426.71	11496.38-11428.11	**cr40**	crTR FRNAb	11071.05	11069.91
**cr4**	crTR_2'5'-p	11426.71	11427.21	**cr41**	crTR_X_2'-5'-b	11059.65	11061.88
**cr5**	2'5'A	11426.71	11430.27	**cr42**	crTR FANA T d	11155.06	11069.64
**cr8**	crTR_J_ FRNAa	11286.94	11286.94	**cr43**	crTR_Xs_dU	10962.97	10963.31
**cr9**	crTR_J_2'-5'-d	11251.00	11249.34	**cr44**	diOMe-CRISPR-b	11323.28	11322.38
**cr10**	crTR_J_2'-5'-o	11251.00	11249.34	**cr45**	2'-F-4'-OMe-CRISPR-b	11251.06	11250.44
**cr11**	crTR FANAj	11370.94	11370.98	**cr46**	crTR FANA U a	11064.93	11069.62
**cr12**	crTR_J_FANAC	11574.77	11541.12	**cr47**	crTR FANA T a	11107.00	11105.96
**cr14**	crTR_I_2'-5'-k	11426.8	11425.30	**cr48**	crTR_X_2'-5'-a	11059.31	11058.22
**cr15**	crTR_I_2'-5'-j	11426.8	11425.40	**cr49**	diOMe-CRISPR-a	11191.11	11190.28
**cr16**	crTR_I_FANAa	11526.80	11526.78	**cr50**	2F4OMe-CRISPR-a	11155.00	11154.28
**cr17**	crTR_J_2'-5'-e	11251.15	11250.07	**cr51**	crTR FRNAa	11065.00	11064.33
**cr18**	crTR_J_2'-5'-f	11251.21	11251.13	**cr52**	crTRFRNAc	11062.98	11062.12
**cr19**	crTR_J_2'-5'-g	11251-11426.8	11250.93-11426.8	**cr53**	crTR FANA U d	11070.91	11069.64
**cr20**	crTR_J_2'-5'-h	11251-11426.8	11251.93-11426.8	**cr54**	crTRFANAF	11103.00	11102.48
**cr21**	crTR_MJ_2'-5'-i	11426.8	11447.7 (+Na)	**cr55**	crTRFANAF2		
**cr22**	crTR_J_FANa	11674.86	11673.94	**cr56**	crTRFANAFS1	11567.02	11567.02
**cr23**	crTR FANAj	11370.94	11370.98	**cr57**	crTRFANAFB1	10934.89	10933.22
**cr24**	crTR_J_FANAD	11412.85	11381.35	**cr58**	crTRFANAh	11328.81	11344.37
**cr25**	crTR_I_FANAb	11454.84	11454.76	**cr59**	crTR FANA T b	11112.92	11111.89
**cr26**	crTR_J_FANAb	11440.98	11441.06	**cr60**	crTRFANAG	11184.88	11232.58
**cr27**	FR/FA3	11430.87	11429.88	**cr61**	crTR FANAC	11094.93	11093.99
**cr28**	FR/FA1	11426.83	11425.32	**cr62**	crTR FRNAe	11244.81	11200.51
**cr29**	FR/FA5	11458.87	11456.91	**cr63**	crTR FRNAf	11184.88	11185.01
**cr30**	FR/FA2	11246.92	11245.90	**crEG7**	89-2 2′-F	12215.38	12214.43
**cr31**	crTR-alt1	11221.00	1122.31	**crEG8**	2′OMe	idt	idt
**cr32**	crTR-alt2	11155.05	11156.54	**crEG9**	84-4 dT	12207.25	12206.57
**cr33**	crTR-alt3	11201.01	11203.21	**crEG10**	85-3 2′-F,4′-OMe	12275.24	12275.12
**cr35**	crTR_S_FRNAa	11107.04	11106.17	**crEG11**	85-4 diOMe	12299.30	12299.51
**cr36**	crTR_S_2'-5'-m	11091.11	11090.09	**crEG12**	97-2 FANA	12215.38	12213.75

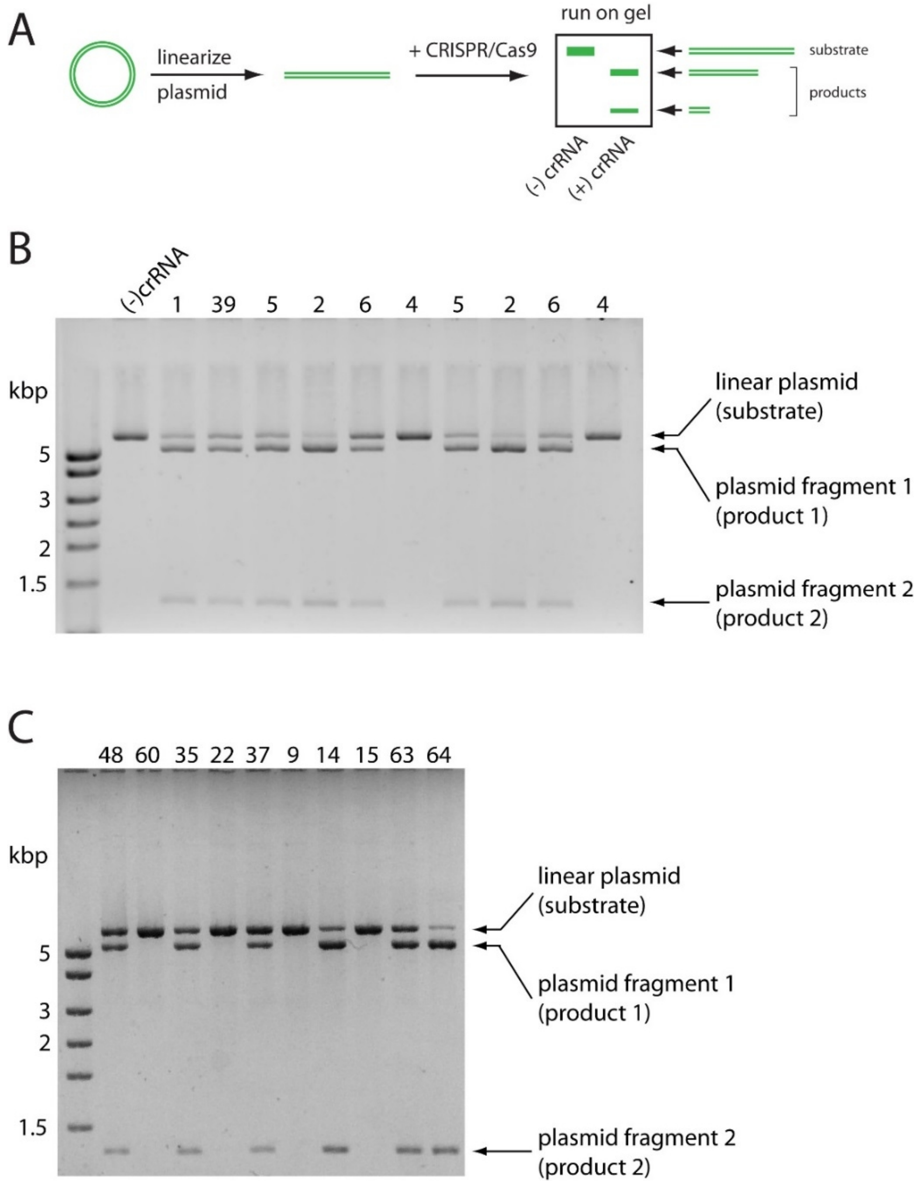

Figure S1. *In Vitro* Cas9 Cleavage Assay. (**A**) Schematic of *in vitro* cleavage of a linearized plasmid. (**B**-**C**) Representative cleavage assay data. crRNA numbers are shown above, base-pair ladder shown to the left, and reaction substrates and products are shown to the right. Gels were quantified with ImageJ software for graphical presentation in main figures.

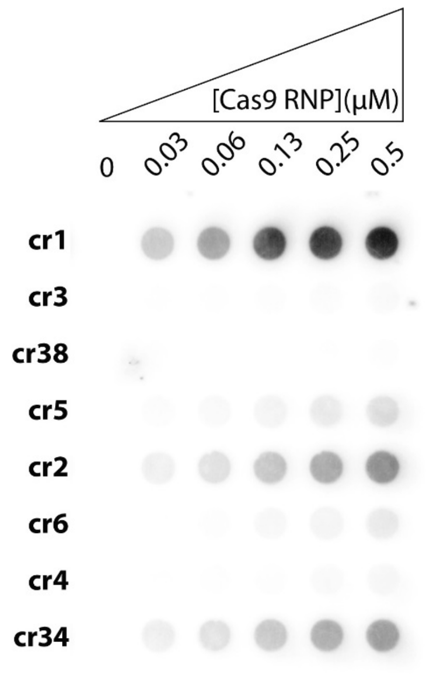

Figure S2. Representative dot-blot data for Cas9 RNP binding to radiolabeled double-stranded target DNA substrate. crRNAs tested are indicated to the left and Cas9-tracrRNA (RNP) concentration is shown above.
